# Cutaneous Focal Mucinosis Successfully Treated With Superpulsed Carbon Dioxide Laser

**DOI:** 10.1155/carm/8484405

**Published:** 2026-06-22

**Authors:** Maryam Hekmat, Arezou Hosseini, Babak Shirazi Yeganeh, Negin Fazelzadeh Haghighi

**Affiliations:** ^1^ Molecular Dermatology Research Center, Shiraz University of Medical Sciences, Shiraz, Iran, sums.ac.ir; ^2^ Department of Dermatology, Shiraz University of Medical Sciences, Shiraz, Iran, sums.ac.ir; ^3^ Department of Pathology, Shiraz University of Medical Sciences, Shiraz, Iran, sums.ac.ir

**Keywords:** carbon dioxide laser, cutaneous focal mucinosis, minimal-invasive therapy

## Abstract

Cutaneous focal mucinosis (CFM) is a rare dermatological condition characterized by localized mucin deposition in the dermis, presenting as solitary or multiple asymptomatic papules or nodules. Treatment options are limited, with spontaneous resolution occurring in some cases, while others persist or require intervention. Systemic therapies, such as isotretinoin, have variable efficacy, and invasive approaches may not suit all patients. We report a 19‐year‐old male case with persistent bilateral papular lesions in the beard area, diagnosed as CFM via biopsy. Due to the patient’s reluctance to use systemic medication, superpulsed carbon dioxide (CO_2_) laser therapy was employed, resulting in significant lesion resolution without adverse effects. At 6‐month follow‐up, the outcome remained favorable, suggesting laser therapy as a promising, noninvasive alternative for localized CFM. This study suggests that superpulsed CO_2_ laser therapy could be a valuable treatment for CFM, especially for patients who prefer nonsystemic or less invasive options. Further studies are warranted to confirm superpulsed CO_2_ laser therapy efficacy and long‐term outcomes in managing this uncommon condition.

## 1. Introduction

Cutaneous focal mucinosis (CFM) is a primary mucinosis of the skin [1]. This disorder was first introduced by Johnson and Helwig in 1966 as a skin lesion resulting from increased deposition of mucin in the dermis layer of the skin [2]. CFM lesions are slightly more prevalent in men, with a male‐to‐female ratio of 1.4 to 1.0, and the age of onset in most patients ranges between 29 and 60 years old [2, 3]. CFM is divided into two subtypes: solitary CFM and multiple CFM [2]. Multiple CFM is associated with other systemic diseases, including scleroderma, thyroid disease, systemic lupus erythematous, scleromyxedema, and Birt–Hogg–Dube syndrome. In cases of multiple CFM, additional laboratory studies are necessary to rule out other systematic diseases [2, 4–6].

CFM appears as single or multiple asymptomatic, dome‐shaped nodules or papules. The lesions can vary in color, ranging from white to flesh‐colored to red [6]. In some instances, they may show erythema with hyperpigmentation, a white lesion with a blueish hue, hyperpigmentation with a light brownish center, or a lesion with an erythematous base. These lesions typically measure less than 10 × 10 mm. Solitary CFM is more commonly found on the upper extremities and upper back. However, there are no site preferences for multiple CFM [2]. This disorder has several clinical differential diagnoses, including amyloidosis, basal cell carcinoma, dermatofibroma, dermal adnexal cyst, neurofibroma, nevus, epidermoid inclusion cyst, lichen planus, keratosis pilaris, acanthosis nigricans, and seborrheic keratosis. Due to variable clinical presentations of CFM and the numerous differential diagnoses, clinical diagnosis can be challenging, and a skin biopsy is necessary to confirm diagnosis and rule out other conditions [2, 7, 8].

The histologic feature of focal cutaneous mucinosis includes mucin that is not encapsulated and scattered fibroblasts in the upper dermis. Mucin can invade the deeper dermis and, in rare cases, the superficial subcutaneous fat [8]. Mucin stains bluish‐purple with toluidine blue, deep blue with colloidal iron, and blue with alcian blue. The lesion’s borders are not well defined, with peripheral edges blending gradually. The overlying epidermis layer may be atrophic, hypertrophic, or normal [2, 8].

The reason behind mucinosis, particularly solitary CFM, remains unclear but is thought to be a benign reactive process, possibly triggered by localized trauma. Unlike systemic mucinoses, which are linked to autoimmune or metabolic disorders, CFM appears as a solitary or localized lesion without systemic involvement [9]. However, in multiple lesion cases, systemic conditions such as thyroid disease or medication use may also contribute [2].

Treatment options for mucinosis are limited; some cases resolve spontaneously [10], while others may persist or enlarge, requiring intervention. Systemic treatments such as isotretinoin are sometimes considered, but their effectiveness is not well‐proven [2]. In this case, superpulsed carbon dioxide (CO_2_) laser therapy was employed as a noninvasive treatment alternative. Its application suggests that it may be a viable option for managing localized mucin deposition, especially for patients unwilling or unable to undergo systemic treatment.

## 2. Case Presentation

**FIGURE 1:**
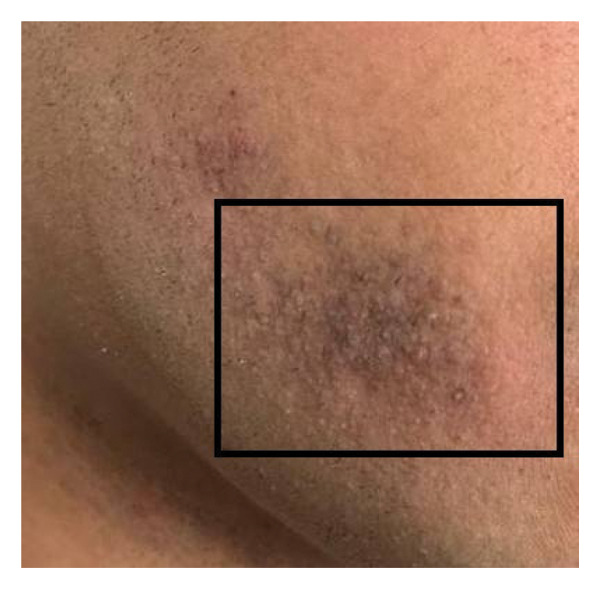
Darkly pigmented papules, before treatment.

A 19‐year‐old male presented to the dermatology clinic at Faghihi Hospital, Shiraz University of Medical Sciences, with bilateral papular lesions in the beard area. The patient pursued medical evaluation due to concerns about the persistence of the lesions and their impact on his appearance. The lesions had developed four years earlier and had remained stable without any associated symptoms. Clinical examination revealed multiple darkly pigmented papules symmetrically distributed in the beard region (Figure 1). Hair growth was significantly reduced in the affected areas, suggesting possible follicular involvement. The patient reported no symptoms such as itching or irritation, had minimal sun exposure, and had no personal or family history of dermatological diseases.

**FIGURE 2:**
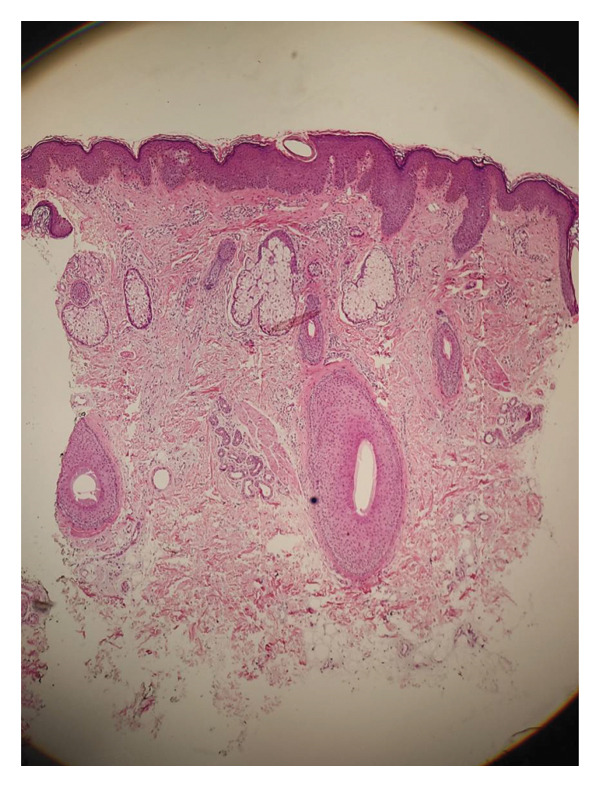
Intact epidermis with mild papillomatosis and an intact basal layer. Dermal sparse perivascular lymphocytic infiltration (× 40 magnification).

**FIGURE 3:**
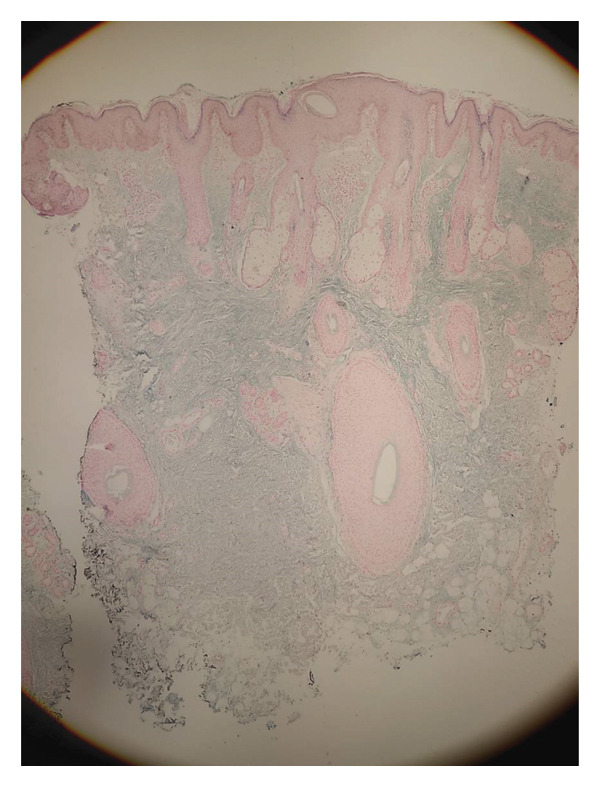
Increased dermal mucin, confirmed by colloidal iron staining. (× 40 magnification).

A biopsy was performed with the differential diagnoses of lichen amyloidosis, lichen planus, keratosis pilaris, acanthosis nigricans, and mucinosis. Histopathological examination showed an intact epidermis with mild papillomatosis and an intact basal layer. The dermis exhibited sparse perivascular lymphocytic infiltration along with increased dermal mucin, which was confirmed by colloidal iron staining. Crystal violet staining for amyloid deposits was negative. Based on these findings, the diagnosis of CFM was suggested (Figures 2 and 3). CBC, fasting blood glucose level, renal, liver, and thyroid function tests, as well as rheumatologic investigations, including ANA, anti dsDNA, anti scl70, C3, C4, ESR, CRP, and HIV antibody were requested, and no abnormality was detected. Considering the possibility of scleromyxedema and systemic involvement, a complete history and physical examination was performed, which were unremarkable. The patient did not agree to perform electrophoresis.

**FIGURE 4:**
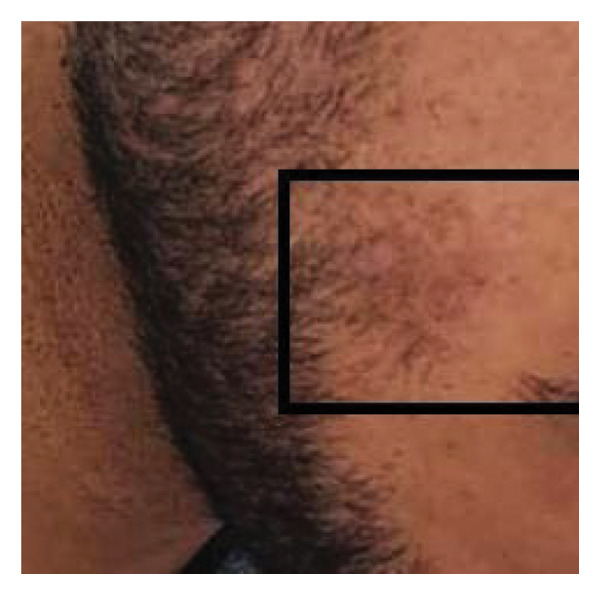
After treatment with superpulsed CO_2_ laser.

Given the patient’s preference for nonaggressive therapeutic options with minimal side effects, we initially prescribed topical pimecrolimus 0.1% twice daily. As no improvement was observed, we discontinued pimecrolimus after two months of treatment. We considered oral isotretinoin as a second treatment modality; however, the patient was reluctant to take systemic medication, leading to the decision to pursue laser therapy instead. After application of topical anesthesia (Xyla‐p cream including 5 g of lidocaine and 5 g of prilocaine in a 30‐g tube) for 30 min, CO_2_ laser treatment at superpulsed mode (fluence: 10 J/cm^2^, pulse duration: 0.1 milliseconds, and fixed spot size: 1 mm, using 2 passes) was applied leading to significant resolution of the lesions after two sessions. Following the laser therapy, the only posttreatment care involved applying a repairing ointment twice daily. The patient tolerated the treatment well and did not experience any adverse effects. At 6‐month follow‐up, the lesions did not recur, and the patient was satisfied with the treatment. Mild postinflammatory hyperpigmentation remained (Figure 4).

## 3. Discussion

The standard management for CFM involves partial or complete excision, often achieved during diagnostic biopsy, as many lesions do not recur after removal [2, 3, 8]. In cases of solitary lesions, surgical excision is both diagnostic and therapeutic, eliminating the need for further intervention [3, 8]. Gutierrs et al. reported a case of focal mucinosis which was presented as an asymptomatic nodule on the right upper shoulder of a 37‐year‐old man. There was no remnant of the lesion after the biopsy. Since no recurrence was observed, they concluded that excisional biopsy alone served as an effective treatment for the lesion [6]. In another case reported by Cohen et al., a 63‐year‐old man referred with pruritic, lichenified, and erythematous lesions and solitary papule on his right upper eyelid, chest, and abdomen for four months. The histopathology confirmed the diagnosis of CFM. The solitary papule was removed with biopsy, and no further treatment was required [2]. However, treatment becomes more challenging in patients with multiple lesions or when residual lesions persist after biopsy. Kim et al. reported a 22‐year‐old man presented with multiple cutaneous mucinosis treated with simple local excision followed by intralesional injection of steroids [11].

Topical therapies, such as corticosteroids and tacrolimus ointment, have shown limited efficacy in CFM [2]. Spontaneous resolution has been documented in some cases, though this remains unpredictable [2]. Given the benign nature of CFM, and a cosmetically sensitive area of involvement in our case, we decided to apply minimally invasive approaches.

In this report, our case presented with bilateral papular CFM lesions in the beard area. Unlike previous reports where lesions resolved after biopsy [3, 8], our patient had residual mucinosis requiring further treatment. To avoid multiple surgical excision sessions, scar formation, and the possible permanent damage to hair follicles following surgery in the beard area, our choice was superpulsed CO_2_ laser therapy, which successfully eradicated the remaining lesion without recurrence. This approach highlights the potential of laser ablation as a noninvasive, effective alternative for CFM, particularly in cases where surgery is undesirable or lesions persist after initial biopsy. However, longer‐term follow‐up is recommended in the future studies to confirm superpulsed laser as a definitive treatment method for local mucinosis, without recurrence.

## Author Contributions

Maryam Hekmat was involved in clinical diagnosis of the case, literature review, and writing the manuscript. Arezou Hosseini was involved in literature review and writing the manuscript. Babak Shirazi Yeganeh was involved in pathological confirmation of the diagnosis and writing the manuscript. Negin Fazelzadeh Haghighi was involved in clinical diagnosis of the case, treatment, and writing the manuscript.

## Funding

No funding was received for this research.

## Ethics Statement

This study was approved by the Ethics committee of Shiraz University of Medical Sciences (IR.SUMS.MED.REC.1404.261).

## Consent

The patient signed written informed consent to permit us use his photograph and publish the article. The personal information of the patient was kept confidential.

## Conflicts of Interest

The authors declare no conflicts of interest.

## Data Availability

All data are available in the main text.
